# Treatment of irritable bowel syndrome with diarrhoea using titrated ondansetron (TRITON): study protocol for a randomised controlled trial

**DOI:** 10.1186/s13063-019-3562-6

**Published:** 2019-08-20

**Authors:** David Gunn, Ron Fried, Rabia Lalani, Amanda Farrin, Ivana Holloway, Tom Morris, Catherine Olivier, Rachael Kearns, Maura Corsetti, Mark Scott, Adam Farmer, Anton Emmanuel, Peter Whorwell, Yan Yiannakou, David Sanders, John Mclaughlin, Kapil Kapur, Maria Eugenicos, Ayesha Akbar, Nigel Trudgill, Lesley Houghton, Phil G. Dinning, Alexander C. Ford, Qasim Aziz, Robin Spiller

**Affiliations:** 1 NIHR Nottingham Digestive Diseases Biomedical Research Centre, University of Nottingham, Nottingham, UK; 2 Nottingham Digestive Diseases Centre, Nottingham University Hospitals NHS Trust, Nottingham, UK; 3 Barts and The London School of Medicine and Dentistry, Queen Mary University of London, London, UK; 4 Wingate Institute of Neurogastroenterology, Queen Mary University of London, London, UK; 5 Clinical Trials Research Unit, Leeds Institute of Clinical Trials Research, University of Leeds, Leeds, UK; 6 Royal Stoke Hospital, University Hospitals of North Midlands NHS Trust, Stoke, UK; 7 University College London Hospital, University College London Hospitals NHS Foundation Trust, London, UK; 8 Wythenshawe Hospital, Manchester University NHS Foundation Trust, Manchester, UK; 9 County Durham and Darlington Foundation Trust, University Hospital of North Durham, Durham, UK; 10 Royal Hallamshire Hospital, Sheffield Teaching Hospitals NHS Foundation Trust, Sheffield, UK; 11 Salford Royal NHS Foundation Trust, Salford Royal University Hospital, Manchester, UK; 12 Barnsley Hospital, Barnsley Hospital NHS Foundation Trust, Barnsley, UK; 13 Western General Hospital Edinburgh, NHS Lothian, Edinburgh, UK; 14 London North West Healthcare NHS Trust, St Mark’s Hospital, London, UK; 15 Sandwell General Hospital, Sandwell and West Birmingham Hospitals NHS Trust, Birmingham, UK; 16 St James’s Hospital, Leeds Teaching Hospitals NHS Trust, Leeds, UK; 17 Discipline of Surgery and Gastroenterology, Flinders Medical Centre, Flinders University, Adelaide, South Australia Australia

**Keywords:** Barostat, Diarrhoea, Irritable bowel syndrome, High-resolution manometry, Ondansetron

## Abstract

**Background:**

Irritable bowel syndrome with diarrhoea (IBS-D) affects up to 4% of the general population. Symptoms include frequent, loose, or watery stools with associated urgency, resulting in marked reduction of quality of life and loss of work productivity. Ondansetron, a 5HT_3_ receptor antagonist, has had an excellent safety record for over 20 years as an antiemetic, yet is not widely used in the treatment of IBS-D. It has, however, been shown to slow colonic transit and in a small randomised, placebo-controlled, cross-over pilot study, benefited patients with IBS-D.

**Methods:**

This trial is a phase III, parallel group, randomised, double-blind, multi-centre, placebo-controlled trial, with embedded mechanistic studies. Participants (*n* = 400) meeting Rome IV criteria for IBS-D will be recruited from outpatient and primary care clinics and by social media to receive either ondansetron (dose titrated up to 24 mg daily) or placebo for 12 weeks. Throughout the trial, participants will record their worst abdominal pain, worst urgency, stool frequency, and stool consistency on a daily basis.

The primary endpoint is the proportion of “responders” in each group, using Food and Drug Administration (FDA) recommendations. Secondary endpoints include pain intensity, stool consistency, frequency, and urgency. Mood and quality of life will also be assessed.

Mechanistic assessments will include whole gut transit, faecal tryptase and faecal bile acid concentrations at baseline and between weeks 8 and 11. A subgroup of participants will also undergo assessment of sensitivity (*n* = 80) using the barostat, and/or high-resolution colonic manometry (*n* = 40) to assess motor patterns in the left colon and the impact of ondansetron.

**Discussion:**

The TRITON trial aims to assess the effect of ondansetron across multiple centres. By defining ondansetron’s mechanisms of action we hope to better identify patients with IBS-D who are likely to respond.

**Trial registration:**

ISRCTN, ISRCTN17508514, Registered on 2 October 2017.

**Electronic supplementary material:**

The online version of this article (10.1186/s13063-019-3562-6) contains supplementary material, which is available to authorized users.

## Background and rationale

Irritable bowel syndrome (IBS), which affects around 10% of the population, accounts for 1.8 million consultations per year in primary care in England and Wales. Around one third of these patients meet criteria for IBS with diarrhoea (IBS-D). Symptoms include frequent, loose, or watery stools with associated urgency, which can severely limit socialising, travelling, and eating out. This can lead to a marked reduction in quality of life and loss of work productivity. When patients with IBS are asked to rank symptoms in order of importance, erratic bowel habit is rated first, followed by abdominal pain and, for those with diarrhoea, urgency [1]. This can often be associated with incontinence, which is socially debilitating, but often under-reported [2].

Current treatments for patients with IBS-D such as loperamide reduce bowel frequency, but do not improve abdominal pain, and often lead to constipation. The lack of effective treatments results in frequent referrals to secondary care, and such patients represent a significant proportion of gastroenterology outpatients.

A previous meta-analysis [3] showed that the 5-hydroxytryptamine-3 receptor antagonists (5HT_3_RAs) alosetron and cilansetron benefited such patients, improving stool consistency, and reducing both frequency and urgency of defaecation. However, these drugs had serious side effects, including constipation in 25% of patients and, rarely, ischaemic colitis (1 in 700). Alosetron was initially withdrawn and now is available in the USA only, through risk evaluation and mitigation strategy (REMS) and is not available in Europe. Cilansetron never came to market, while ramosetron, another 5-HT3 receptor antagonist is only available in Japan where it is licensed for IBS-D, with several good-quality trials confirming its benefit [4, 5].

Ondansetron is a widely used 5HT_3_RA that, unlike alosetron, has not been associated with ischaemic colitis. A pilot randomised, placebo-controlled cross-over trial showed that 5 weeks of ondansetron was effective in improving diarrhoea and urgency [6]. Currently we do not understand exactly how it works, nor can we predict the individual dose required for optimum effect, which varies widely. One key effect we found, also seen with other 5HT_3_RAs [7], was a marked reduction in urgency, which may be important in improving quality of life in patients with IBS-D [8].

### Potential mechanisms of action of 5HT_3_ receptor antagonists

The 5HT_3_RAs slow colonic transit, an effect we found particularly marked in the left colon and the rectosigmoid region of patients with IBS-D, but the underlying mechanism was unclear [6]. Previous studies of the impact of 5HT_3_RAs on human colonic motility [9, 10] showed that the 5HT_3_RAs alosetron and cilansetron increased peri-prandial frequency of colonic contractions, and mean amplitude of contractions in the left colon. We hypothesise that 5HT_3_RAs increase retrograde sigmoid motility, perhaps enhancing “brake” function [11, 12], which would be a novel mode of action. In our pilot study we showed the decrease in urgency correlated directly with the reduction in faecal protease [13], but whether this represents a true causal relationship or just an epiphenomenon is unclear. Faecal proteases have been shown to be increased in IBS-D and, at least in animal models, cause hypersensitivity to rectal distension via activation of protease activated receptors type 2 (PAR2) [14]. We have shown that most faecal proteases are endogenous [13], representing pancreatic enzymes that have escaped degradation by colonic bacteria. We hypothesise that slowing gut transit reduces faecal protease, by allowing time for bacterial degradation, and that this may also contribute to the beneficial effects of ondansetron. This might also improve the anal soreness that is commonly reported by patients with IBS-D. Bile acids have also been shown to sensitise the rectum [15], and elevated faecal bile acids have been identified by several groups in patients with IBS-D [16]. Slowing transit may increase the time for bile acid deconjugation by colonic bacteria, and therefore enhance absorption, but how important this is in reducing rectal sensitivity, compared with the effects on faecal proteases, is unclear.

### 5HT_3_ receptor antagonist sensitivity

We have also shown that individuals vary widely in their responsiveness to ondansetron, explaining why trials using fixed doses of 5HT_3_RAs result in severe constipation in some patients. When patients were allowed to have dose titration we found that constipation was rare, occurring in only 2% of patients. However, the required dose of ondansetron ranged from 4 mg on alternate days to 8 mg three times a day (t.d.s.). The reasons for this variation are unclear but recent evidence suggests that responsiveness to 5HT_3_RAs might be linked to polymorphisms in the genes controlling 5HT synthesis. Serotonin availability in the rectal mucosa is thought to be determined by the activity of the rate-limiting synthetic enzyme tryptophan hydroxylase-1 (TPH-1), which produces serotonin in enterochromaffin cells. A recent small study showed that TPH-1 mRNA levels in rectal mucosa (and thus presumably serotonin synthesis rate) were approximately doubled in responders to another 5HT_3_RA, ramosetron, compared with non-responders, and that this was linked to the TPH-1 genotype [17]. TPH-1 rs211105 minor allele G was found in 44% of non-responders, but only in 4% of responders, indicating that possessing the major allele increases responsiveness to the drug. It was also associated with worse diarrhoea, possibly because of the greater 5HT synthesis.

### Investigational agent

Ondansetron is a potent, highly selective 5HT_3_RA, which blocks 5HT_3_ receptors in the gastro-intestinal tract and in the central nervous system. Ondansetron is currently licenced for use in adults and children for the management of nausea and vomiting induced by cytotoxic chemotherapy and radiotherapy, and for the prevention and treatment of post-operative nausea and vomiting. Constipation is an unintended side effect of ondansetron, which was first shown to slow colonic transit 30 years ago [18–20]. In our pilot study, 120 patients were recruited to a randomised double-blind, placebo-controlled, cross-over trial of ondansetron treatment for IBS-D. Patients were randomised to receive ondansetron (2 mg per day up to 8 mg t.d.s) followed by placebo or placebo followed by ondansetron. Patients began on drug A for a period of 5 weeks, then underwent a washout period of 2–3 weeks, and then commenced drug B for 5 weeks. The primary outcome measure for the study was the difference in average stool consistency in the last 2 weeks of treatment of ondansetron versus placebo, and this showed a highly significant improvement with active treatment. We also showed significant benefits for both urgency and stool frequency, with associated slowing of whole gut transit. Despite having limitations, the results of this pilot study were very encouraging, supporting our clinical experience of the benefits of ondansetron.

## Objectives

The primary objective is to determine the effectiveness and safety of ondansetron in patients with symptoms of IBS-D, including urgency, looseness of stool, frequency of defaecation, and abdominal pain. The trial also aims to understand further the mode of action of ondansetron in these patients. More specifically, we will examine the role of rectal sensitivity and compliance, faecal bile acids and proteases, post-prandial sigmoid motility, and genetic variation of serotonin synthesis, both in terms of symptom generation in IBS-D and in responsiveness to ondansetron. This will be achieved by performing mechanistic studies within the clinical trial to determine if changes in symptoms are correlated with changes in these biomarkers.

### Trial design

**Fig. 1:**
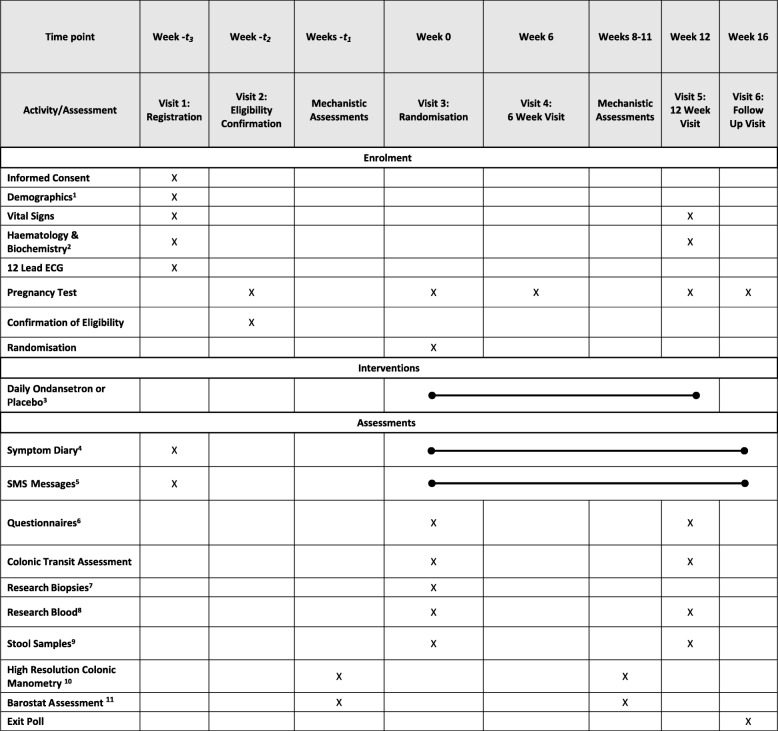
Schedule of enrolment, interventions and assessments throughout the TRITON study. ^1^ Date of birth, National Health Service (NHS) number, address, telephone number (if consented to text messages), smoking history, height, weight. ^2^ White blood cell count (WBC), full blood count (FBC), liver function tests (LFTs), urea and electrolytes (UEs), C-reactive protein (CRP). ^3^ Titrated to optimum dose during the first 2 weeks. ^4^ Recording worst abdominal pain (0–100), worst urgency (0–100), number of investigational medicinal product (IMP) taken, use of loperamide, stool consistency of each stool passed, and relief from irritable bowel syndrome (IBS) symptoms at the end of each week. ^5^ Asking if they have passed a stool type 6 or 7, and what their worst abdominal pain score was that day. ^6^ At baseline only, Physical Symptoms Questionnaire (PHQ)-12; at baseline and visits, IBS Severity Scoring System (IBS-SSS), Short-form Leeds Dyspepsia Questionnaire (SF-LDQ), Hospital Anxiety and Depression Scale (HADS), and IBS Quality of Life Questionnaire (IBS-QOL). ^7^ Six high-rectal biopsies taken either during mechanistic assessment or at visit 3. ^8^ Whole blood (5 ml) at visit 3; serum (5 ml) at visits 3 and 5. ^9^ Four aliquots obtained and frozen at home prior to visit. ^10^ Performed at University of Nottingham & QMUL only (*n* = 40); patients will receive additional payment for participation. ^11^ Performed at Nottingham/Leeds/QMUL/UCL only (*n* = 80); patients will receive additional payment for participation

TRITON is a multi-site, parallel group, randomised, double-blind, placebo-controlled trial with embedded mechanistic studies within selected sites. Our aim is to determine the superiority of ondansetron compared with placebo. In total, 400 patients with IBS-D will be randomised 1:1 to receive either ondansetron or placebo. Both treatments will be administered in oral doses of between 4 mg every third day and 24 mg daily for 12 weeks. Dose titration will be undertaken in the first 2 weeks of the trial to avoid constipation, which at a standard dose occurs in one quarter of patients. The primary outcome will be assessed over the 12 weeks post randomisation. Secondary and safety outcomes will be measured up to 16 weeks following randomisation. See Fig. 1 for an overview of the trial procedures. The Standard Protocol Items: Recommendations for Interventional Trials (SPIRIT) checklist is provided as an Additional file 1.

## Methods

### Trial setting

This trial will be performed initially at 18 sites in the UK. All sites will be required to recruit patients, perform all necessary protocol assessments, and offer patients the opportunity to take part in the whole-gut transit assessment and the blood and stool collection. In addition, four of the sites will perform the mechanistic assessments for the trial. Patients at all sites will be offered an opportunity to take part in the mechanistic studies but will be required to travel to one of the specified sites for these assessments.

### Eligibility criteria

#### Inclusion criteria

Patients must fulfil all of the following criteria:

- Meet Rome IV criteria for IBS-D (see Table 1)
- Age ≥ 18 years
- Have completed standardised workup to exclude
  - Microscopic colitis (colonoscopy or flexible sigmoidoscopy with colonic biopsies)
  - Bile acid diarrhoea (selenium homocholic acid taurine (SeHCAT) > 10%, C4 < 19 ng/ml or failed to respond to 1 week trial of a bile acid binding agent (colestyramine 4 g t.d.s., colesevelam 625 mg t.d.s. or equivalent))
  - Lactose malabsorption (suggested but not mandated negative lactose breath hydrogen test, negative clinical challenge or failure to respond to lactose-free diet)
  - Coeliac disease (confirmed by tissue transglutaminase (tTG) or duodenal biopsy)
- Patients of childbearing potential or with partners of childbearing potential must agree to use methods of medically acceptable forms of contraception during the trial and for 90 days after completion of trial medication
- Women of childbearing potential must test negative for pregnancy within 72 h of confirmation of eligibility
- Weekly average worst pain score ≥ 30 on a 0–100-point scale
- Stools with a consistency of 6 or 7 on the Bristol Stool Form Score (BSFS) for 2 or more days per week

**Table 1:** Rome IV diagnostic criteria for irritable bowel syndrome with diarrhoea

Meet Rome IV criteria for IBS-D for the past 3 months:	
- Recurrent abdominal pain at least weekly	
- Pain is associated with two or more of the following; related to defaecation, associated with a change in frequency of stool, associated with a change in form of stool	
- Symptoms onset at least 6 months prior to diagnosis	
- Abnormal stools are loose (BSFS 6 or 7) in > 25% of cases but are hard (BSFS 1 or 2) in < 25% of cases	

#### Exclusion criteria

Patients must not fulfil any of the following criteria:

- Gastrectomy
- Intestinal resection;
- Other known organic gastrointestinal diseases (e.g. inflammatory bowel disease (Crohn’s disease, ulcerative colitis));
- Unable or unwilling to stop restricted medication including regular loperamide, antispasmodics (e.g. buscopan, mebeverine, peppermint oil, alverine citrate), eluxadoline, tricyclic antidepressant doses > 30 mg/day or other drugs likely in the opinion of the investigator to alter bowel habit. These medicines should be discontinued for a 7-day washout period prior to registration;
- QTc interval ≥ 450 msec in men or ≥ 470 msec in women (assessed within the last 3 months by electrocardiogram (ECG));
- Previous use of ondansetron for chronic symptoms, or contraindications to ondansetron;
- Pulse, blood pressure, laboratory-tested blood values outside the normal ranges according to the site’s local definition of normal (assessed within the last 3 months). Note minor rises in alanine aminotransferase (ALT) (< 2 × upper limit of normal) will be acceptable, but the patient’s General Practitioner (GP) will be informed if ALT remains elevated at the end of the trial;
- Women who are pregnant or breastfeeding;
- Patients currently or previously participating in a trial of an investigational medicinal product (IMP) in the previous 3 months, where the use of the IMP may cause issues with the assessment of causality in this trial;
- Patients who have started or who have had alterations to their dosage of selective serotonin re-uptake inhibitors or tricyclic antidepressants in the last 3 months, or who will have their dosage changed during the trial;
- Patients currently taking and unwilling or unable to stop taking apomorphine or tramadol (which interact with ondansetron);
- Patients with only stools of consistency 7 on the BSFS for 7 days a week.

Patients taking QT-prolonging or cardiotoxic drugs will be reviewed by the local Principal Investigator (PI) to determine whether they are suitable for the trial.

### Intervention

Eligible patients will be randomised in equal proportions to receive either over-coated capsules of ondansetron 4 mg or over-coated placebo, for 12 weeks. The dose of the IMP will be individually titrated during the first 2 weeks by calls from the research team on alternate days, aiming for a stool consistency type 3–5 on the BSFS.

#### Adherence

Patients will receive face-to-face adherence reminders on each trial visit, as well as the phone calls during the 2-week titration period. Any remaining IMP capsules are counted at visits 4 and 5.

#### Dose titration

Since the optimum dose varies widely from 4 mg on alternate days up to 8 mg t.d.s., we will start all patients on 4 mg daily and after 2 days contact them to adjust the dose, thus avoiding the complication of constipation. If stool consistency remains loose, patients will be asked to increase their dose in 4-mg steps every 2 days up to the maximum of 8 mg t.d.s. If stools become hard or there is no bowel movement on day 2, they will be asked to stop the drug for 1 day and recommence at a lower dose changing from 4 mg daily to 4 mg on alternate days. If stools still remain hard or infrequent, patients will be asked to reduce their dosage to 4 mg every third day. Patients will discontinue the trial in the unlikely event that their stool remains hard even at this low dose.

### Outcomes

#### Primary outcome measure

The primary outcome measure is to ascertain whether 12 weeks of ondansetron increases the FDA-defined responder rate (in relation to abnormal defaecation and abdominal pain) compared with placebo. A responder is a patient who records both a reduction in pain intensity (≥ 30% decrease from baseline in weekly average worst daily pain) and improvement in stool consistency (≥ 50% decrease in the number of days per week with ≥ 1 loose stool ((BSFS) [21] 6 or 7)) for at least 6 weeks of the 12-week treatment period.

#### Secondary outcome measures

Secondary outcome measures will include:

- Stool consistency and abdominal pain (measured by diary and daily text message);
- Stool frequency, urgency of defaecation, and use of rescue medication (defined as the use of loperamide) over 12 weeks of treatment and the answer to the question “Overall, have you had satisfactory relief from your IBS symptoms in the past week?” (measured by diary);
- The following will also be assessed at the beginning and the end of the trial: IBS symptom severity (measured by the Irritable Bowel Syndrome Severity Scoring System (IBS-SSS) [22]), dyspepsia (using the Short Form Leeds Dyspepsia Questionnaire (SF-LDQ) [23]), quality of life and mood (using the Irritable Bowel Syndrome Quality of Life (IBS-QOL) [24] and Hospital Anxiety and Depression Scale (HADS) [25] questionnaires), and somatic symptoms (using the Patient Health Questionnaire 12 Somatic Symptoms (PHQ-12) [26] questionnaire);
- Stool frequency, stool consistency, urgency, abdominal pain, and adverse events (AEs) assessed 4 weeks after the end of treatment to determine if there are any persisting effects.

#### Mechanistic outcome measures

The trial will evaluate the possible mechanisms underlying any changes in the primary and secondary endpoints. The effect of ondansetron on whole gut transit will be measured at baseline and 12 weeks (*n* = 400), using radio-opaque markers and an abdominal x-ray as previously described [27]. High-resolution manometry will be performed at baseline and after 8–11 weeks of treatment at two centres (*n* = 40) to assess whether ondansetron decreases the number of high-amplitude propagating contractions and increases the percentage time occupied by cyclical retrograde propagated contractions [11]. Barostat assessment will be performed at baseline and after 8–11 weeks of treatment at four centres (*n* = 80, some of whom may also undergo manometry), in order to assess if ondansetron increases rectal compliance and decreases sensitivity (manifested as increased pressure thresholds for pain and urgency). Serum will be sampled to genotype patients for polymorphisms in the *TPH1* gene (*n* = 400), and correlation tested between these and the mucosal 5HT and TPH-1 mRNA obtained from high-rectal biopsies (*n* = 80), final ondansetron dose, and responder status, to see whether this predicts sensitivity and/or response to ondansetron. Finally, stool sample analysis (*n* = 400) will be used to assess whether ondansetron reduces total faecal bile acid and tryptase concentrations, and correlation will be tested between these and any changes in urgency.

### Participant timeline

#### Visit 1

Potential trial candidates will attend their first visit for registration and consent by the PI or delegate. If required, further tests to exclude diagnoses other than IBS-D will be arranged. These include a SeHCAT scan, serum C4 level, or a 1-week trial of a bile-acid binding agent to assess for bile acid malabsorption (unless done within the last 5 years), and colonoscopy (unless done within 2 years, or 5 years if they also currently have normal calprotectin) to assess for microscopic colitis. Baseline serum blood tests, vital signs, demographics (date of birth, gender, ethnicity, and smoking history) and an ECG will be obtained. Current medications will be reviewed, and those unable to discontinue drugs likely to alter bowel habit will be unable to enter the trial. Patients on QT-prolonging drugs and cardiotoxic drugs will be reviewed by the PI for suitability for the trial, as ondansetron may increase the risk of QT prolongation and arrhythmias. Eligible and consenting patients are registered and allocated a unique trial ID and data collected will be link anonymised.

All patients will be asked to complete a 2-week daily diary recording stool frequency, the consistency of each stool (using the BSFS), worst abdominal pain (on a scale of 0–100), worst bowel movement urgency (on a scale 0–100), and if they have used loperamide that day. In addition, patients have the option to be sent two automated text messages each day. The first will ask the patient if they have passed a stool of consistency 6 or 7 on the BSFS. They will need to reply with either a yes or no. The second text message will ask what their worst abdominal pain score was that day. The patient must respond with a number from a scale of 0–100 (where 0 is no pain and 100 is the worst imaginable pain).

#### Visit 2

The patient will return 2 weeks later to confirm eligibility. The diary will confirm they have had stool consistency BSFS 6–7 for more than 2 days a week and do not have only BSFS 7 for 7 days per week, and a weekly average worst pain score ≥ 30. Patients who consent to the whole gut transit study will be dispensed Transit-Pellet capsules containing markers, and the abdominal x-ray appointment will be confirmed for the morning of visit 3. Patients will take the Transit-Pellet capsules for 6 days prior to visit 3. Patients who have consented to one or both mechanistic studies will have appointments arranged for baseline assessment prior to visit 3.

#### Visit 3

On visit 3, patients will undergo a pregnancy test if applicable, whole gut transit assessment by abdominal x-ray (if they have consented), rigid sigmoidoscopy (if they have consented), completion of baseline questionnaire booklet (including IBS-SSS, SF-LDQ, HADS, PHQ-12, and IBS-QOL questionnaires), and collection of stool, whole blood, and serum samples (if they have consented). Patients will then be randomised and given a 6-week patient diary and the trial medication in accordance with their blinded randomisation allocation. Patients will be asked to record the following on a daily basis: stool frequency, consistency of each stool, worst abdominal pain experienced that day, worst bowel movement urgency, number of trial medication capsules taken, and whether they have used loperamide that day. Every week the diary will ask whether they feel that they have had satisfactory relief from their IBS symptoms that week. If they agree, the patient will continue to receive two text messages each day for the next 6 weeks, asking if they have passed a stool of a consistency of 6 or 7 that day, and what their worst abdominal pain was that day.

During the first 2 weeks patients will be contacted every 2 days by the local site team to discuss bowel habit. The dose of ondansetron or placebo will then be titrated as required. Additional guidance on dose titration will be given to each trial site in a standard operating procedure, and to the patient in a dose titration instruction leaflet. A check for serious adverse events (SAEs) will be performed during each telephone call. The steady dose to be taken forward for the remainder of the trial will be confirmed in week 2, although this may be altered during the 12 weeks if required, to avoid constipation.

#### Visit 4

Patients will return for their fourth visit at 6 weeks of the trial treatment period. Diaries will be collected, and the investigator will ask whether any reportable AEs have occurred since the last visit. A pregnancy test will be taken and concurrent medications will be reviewed to ensure these do not interfere with the trial medication. A further 6-week patient diary, trial medication, and Transit-Pellet capsules (for use 6 days prior to visit 5) will be dispensed. Patients who have consented to mechanistic studies will have appointments confirmed and these will take place between 8 and 11 weeks of treatment. Daily text messages will be sent for a further 6 weeks to those patients who agree.

#### Visit 5

Patients will return for visit 5 after 12 weeks on the trial medication. A pregnancy test will be taken, concurrent medication will be reviewed to ensure that these do not interfere with the trial medication, and the investigator will ask whether any reportable AEs have occurred since the last visit. Unused medication and completed patient diaries will be collected. The abdominal x-ray to assess whole gut transit will be performed in consenting patients. Serum and stool samples will be collected from consenting patients, and all patients will complete the 12-week questionnaire booklet, including the IBS-SSS, SF-LDQ, HADS, and IBS-QOL questionnaires. Patients will be issued with a follow up diary and will continue to respond to text messages for a further 4 weeks.

#### Visit 6

Patients will then return for the sixth and final visit, where the diary will be collected and the investigator will ask whether any reportable AEs have occurred since the last visit.

### Sample size

TRITON plans to recruit 400 patients from up to 24 sites across England and Scotland. This will provide 90% power at 5% significance to detect a 15% absolute difference between the randomised groups in the proportion of patients achieving the FDA-recommended [28] endpoint of a weekly response for pain intensity and stool consistency for at least 6 weeks of the 12-week treatment period. This difference (15%) is considered to represent the minimum clinically important difference. We have assumed a placebo response rate of 17%, as recently reported using this endpoint and allowed for a 15% attrition rate.

#### Whole gut transit

Our previous study using the same radio-opaque marker technique as we propose to use showed ondansetron increased whole gut transit time by a mean (95% CI) of 10 (6–14) h. Using 200 patients per group gives 90% power to detect a change of 0.7 h. The larger numbers will also give us the power to test correlation with other endpoints.

#### High-resolution left-sided colonic manometry

Previous studies with the closely related 5HT_3_RA alosetron showed an increase in motility index compared with placebo, with a mean (standard deviation (SD)) of 1.0 (1.2) [29], indicating we would have 80% power to detect a standardised effect size of 1 with 17 patients. We will aim for 20 patients on each treatment to allow for dropouts i.e. 40 each undergoing two studies, a total of 80 high-resolution manometry (HRM) studies.

#### Rectal compliance and sensitivity

Previous studies [29] with alosetron showed an increase in compliance from 5.9 (SD 1.3) to 9.8 (SD 1.2) ml/mmHg in 22 patients. We propose to study more patients to test correlation with symptoms, which typically requires much larger numbers, so we will aim to study 40 patients on each treatment.

### Recruitment

Patients with IBS-D will be identified at recruiting sites from outpatient clinics and lists of patients that have previously consented to be contacted for information on upcoming research studies by local investigators and research nurses. Potentially eligible patients with IBS-D will also be identified by primary care general practices and local pharmacies, working either as patient identification centres (PIC), or a source of trial advertising. The TRITON trial will also be advertised using posters and leaflets in electronic and paper form and distributed to relevant locations outside of the secondary care setting and will advise patients to visit the trial website for further information. Patients will be screened against the aforementioned inclusion and exclusion criteria and will be provided with trial information.

### Randomisation

Randomisation will be performed on a 1:1 basis to receive either ondansetron or placebo, and each patient will be allocated three bottles of trial medication, each with a unique IMP kit code. Minimisation will be used, in order to ensure treatment groups are well-balanced. The stratification factors are registering site and whether the patient has undergone mechanistic assessments.

### Blinding

As the trial is double-blind, neither the patient nor those responsible for their care and evaluation (treating team and research team) will know the allocation or coding of the treatment allocation. This will be achieved by identical packaging and labelling of both the over-encapsulated ondansetron and matched placebo. Each bottle of ondansetron/placebo will be identified by a unique kit code. Randomisation lists containing kit allocation will be generated by the safety statistician at the Clinical Trials Research Unit (CTRU) and sent to the clinical supply company, which will produce the kits and the code-break envelopes. Management of kit codes on the kit logistics application, which is linked to the 24-h randomisation system, will be conducted by the CTRU safety statistician in addition to maintaining the back-up kit-code lists for each site.

Access to the code break envelopes will be restricted to the safety statistician and designated safety team. Code breaks will be permitted in emergency situations, where treatment allocation knowledge is needed to optimise treatment of the patient. Any unblinded interim reports provided to the Data Monitoring and Ethics Committee (DMEC) will be provided by the CTRU safety statistician and the reports will be securely password-protected.

### Data collection methods

#### Primary outcome method

##### Responders

According to the FDA definition [28], a responder is a patient who records both a reduction in pain intensity (≥ 30% decrease from baseline in weekly average worst daily pain) and improvement in stool consistency (≥ 50% decrease in the number of days per week with ≥ 1 loose stool of BSFS [21] 6 or 7) for at least 6 weeks of the 12-week treatment period, which is recorded in the daily diary and text messages.

#### Secondary outcome methods

Daily diary entries will record stool consistency, worst abdominal pain, stool frequency, worst urgency of defaecation, use of rescue medication over 12 weeks of treatment, and the answer to the question “Overall, have you had satisfactory relief from your IBS symptoms in the past week?”. The same diaries will be used between visits 5 and 6 to determine if there are any persisting effects of the treatment. Questionnaire booklets at visits 3 and 5 will record IBS-SSS, SF-LDQ, IBS-QOL, and HADS.

#### Mechanistic outcome methods

Whole gut transit (*n* = 400) will be assessed at visits 3 and visit 5 by ingestion of radio-opaque markers 6 days before an abdominal x-ray, as previously described [27]. Serum will be sampled (*n* = 400) at visit 3 to genotype patients for polymorphisms in the *TPH1* gene. Stool samples (*n* = 400) will be collected and frozen by patients in their homes prior to randomisation and at visit 5 to assess for total faecal bile acid and tryptase concentrations.

High-resolution manometry (*n* = 40) will be performed prior to randomisation and between 8 and 11 weeks of treatment, at two centres. Patients will fast overnight, then receive a tap water enema at 37 °C prior to endoscopically siting the manometry catheter, which is clipped to the mucosa of the splenic flexure. After 30 min of rest, the manometer will start recording for 4 h. At 2 h the patient will be given a meal to stimulate colonic contractions.

Barostat assessment (*n* = 80) will be performed prior to randomisation and between 8 and 11 weeks of treatment, at four centres. Patients will fast overnight, then receive a tap water enema at 37 °C prior to digital insertion of a barostat rectal compliance balloon. The bed will be tilted 15° head down to reduce abdominal visceral pressure on the rectum. After calibration the balloon will be inflated in increments of increased pressures, during which time patients will respond with their corresponding sensation of no sensation, first sensation, desire to defecate, urgency, discomfort, or pain. If the sensation of pain is reached the balloon is immediately deflated.

Six high-rectal biopsies will be obtained per patient (*n* = 80) either by endoscopy after siting of the colonic manometer or via rigid sigmoidoscopy after barostat assessment or at visit 3. Four biopsies will be snap-frozen and two will be placed in RNA later prior to freezing at − 80 °C before being analysed for mucosal 5HT and TPH-1 mRNA.

### Data analysis

#### General considerations

All hypothesis tests will be two-sided with a 5% significance level. Methods to handle missing data are described for each analysis. Analysis and reporting will be in line with Consolidated Standards of Reporting Trials (CONSORT) [30]. As TRITON is a double-blind trial, the trial statistician will be blinded to treatment group allocation throughout the trial, until the database has been locked and downloaded for final analysis. Only the safety statistician, supervising trial statistician, back-up safety statistician, and authorised unblinded individuals at the CTRU will have access to unblinded treatment group allocation prior to final analysis.

#### Frequency of analyses

Outcome data will be analysed once only at the final analysis, although statistical monitoring of safety data will be conducted throughout the trial and reported at agreed intervals to the DMEC. Final analysis will take place 16 weeks after the last patient is randomised.

#### Endpoint analysis

All analyses will be conducted on the intention-to-treat population, defined as all patients randomised regardless of non-compliance with the intervention. The primary endpoint will be analysed per-protocol to indicate whether results are sensitive to the exclusion of patients who violated the protocol (e.g. those patients randomised but subsequently found to be ineligible). Primary and secondary analysis will be performed blinded to random allocation. Outcome measures will be analysed by regression models appropriate to the data type. Such analyses will be adjusted for randomisation minimisation factors: site, completion of manometry assessment, barostat assessment, and baseline values where applicable, age, and gender. Baseline characteristics will be summarised by randomised group.

#### Primary analysis

The primary analysis will compare the difference between treatment groups in the proportion of patients achieving the FDA-recommended endpoint at 12 weeks post-randomisation, using a logistic regression model adjusted for minimisation, age, and gender. Any missing data will be assumed missing at random (MAR) and imputed for the primary analysis. Odds ratios and corresponding 95% confidence intervals will be presented.

Sensitivity will be analysed to assess the impact of missing data on the treatment effect. This will include complete case analysis and alternatives to multiple imputation (e.g. pattern mixture modelling) if missing patterns suggest data are missing not at random.

#### Secondary analyses

The difference between the treatment groups in the proportions of patients with satisfactory relief of IBS symptoms at 12 weeks post-randomisation will be analysed using logistic regression models, adjusting for minimisation, baseline values, age, and gender. Odds ratios and corresponding 95% confidence intervals will be presented. Any missing data will be assumed MAR and imputed.

The differences between the two treatment groups in the continuous secondary endpoints at 12 weeks post-randomisation will be analysed using linear regression models, adjusted for the minimisation variables, baseline values where applicable, age, and gender. These endpoints are urgency of defaecation over the last month, stool frequency over the last month, number of days per week with at least one loose stool (BSFS > 5) over the last month, average stool consistency, number of days rescue medication used over 12 weeks, abdominal pain score, HADS depression and anxiety scores, SF-LDQ score, IBS-QOL score and subscales, and PHQ-12 and IBS-SSS severity scores. Treatment estimates and corresponding 95% confidence intervals will be reported. Any missing data will be assumed MAR.

The differences between the treatment groups in stool frequency, abdominal pain, and urgency of defaecation post-treatment, over weeks 13–16 post-randomisation, will be analysed using a linear regression model adjusted for minimisation factors, baseline values, and relevant baseline factors. Treatment estimates and corresponding 95% confidence intervals will be reported. Any missing data will be assumed MAR.

Exploratory analyses on the daily measurements (worst abdominal pain, loose stools, number of stools passed, consistency of stool, worst urgency, and use of loperamide) will be carried out using repeated measures models, which incorporate correlation between measurements from the same patient. SAS software version 9.4 will be used in the analyses of primary and secondary endpoints.

#### Safety analyses

All patients who receive at least one dose of trial treatment will be included in the safety analysis set. The number of patients reporting a SAE (up to 28 days after the last dose of treatment) and details of all SAEs will be reported for each treatment group. The number of patients withdrawing from trial treatment will be summarised by treatment arm, along with reasons for withdrawal. All safety analyses performed prior to final analysis will be undertaken by the safety statistician (rather than the trial statistician), thus ensuring that the trial team remain blinded.

#### Subgroup analyses

No subgroup analyses are planned.

#### Mechanistic studies

Mechanistic studies will be analysed blinded to the intervention allocation, by the site research fellow under supervision of the Chief investigator and local supervising PIs. The differences between treatment groups in changes in whole gut transit times, colonic motility measures (percent time of cyclical retrograde contractions and high-amplitude propagating contraction (HAPC) frequency), rectal compliance, and thresholds for urgency and pain measured using the barostat, faecal bile acid concentrations, and faecal tryptase will each be assessed by linear regression models. In addition, exploratory mediator analyses will explore whether treatment effects, in terms of changes in urgency or pain, are mediated through changes in faecal bile acids or protease. Exploratory subgroup analysis (tests of interactions) will be performed to investigate the effect of the presence of each specified single nucleotide polymorphism allele on response to treatment, using logistic regression with addition of an interaction term for the allele and treatment.

### Adverse events

An AE is any untoward medical occurrence (including deterioration of a pre-existing medical condition) in a patient or clinical trial patient administered a medicinal product, and which does not necessarily have a causal relationship with this product. The occurrence of reportable AEs will be recorded at visits 4, 5, and 6. At each visit the research nurse will complete the AE checklist to determine if the patient has suffered with any of the expected AEs. Only the confirmation of occurrence and corresponding severity will be recorded.

### Committees

#### Trial Management Group

A Trial Management Group (TMG) will be convened including the Chief Investigator, co-investigators and identified key collaborators, the trial statistician, and trial manager. Notwithstanding the legal obligations of the Sponsor and Chief Investigator, the TMG will have operational responsibility for the conduct of the trial. The TMG will meet quarterly as a minimum, and will be responsible for protocol completion, case report form (CRF) development, and monitoring of screening, recruitment, treatment, and follow-up procedures.

#### Data Monitoring and Ethics Committee

A DMEC will be convened to monitor data collected during the trial and make recommendations to the Trial Steering Committee (TSC) on whether there are any ethical or safety reasons as to why the trial should not continue. It will consist of an independent Chair, an independent statistician, and an independent clinician.

#### Trial Steering Committee

A TSC will be convened with an independent majority. Participants will include as a minimum, an independent Chair, an independent statistician, an independent clinician, a patient and public involvement (PPI) representative, the Chief Investigator, the Sponsor’s representative, and other members of the TMG as required to update on trial progress. The role of the TSC will be to provide overall supervision of the trial progress and advice on operational issues as necessary to the TMG. The TSC will meet annually as a minimum.

## Discussion

Our pilot study has shown that ondansetron provides an opportunity to help patients with IBS-D, not only with diarrhoea, but also with urgency, frequency of defecation, and bloating. When patients with IBS-D are asked about their concerns, erratic unpredictable bowel habit and urgency in particular, are rated as having the greatest impact on quality of life, so improvement in urgency and stool frequency may be of considerable value. However, abdominal pain is a key part of IBS, so we have used the combined FDA-recommended outcome measure, which includes both reduction in pain and improvement of diarrhoea as our primary endpoint, rather than just stool consistency, as in the pilot.

This larger clinical trial aims to confirm the effectiveness of ondansetron in managing IBS-D and, by mechanistic assessments, shed light on both the pathophysiology of the condition and the mode of action of ondansetron in this population. With greater understanding of the condition, we hope this will allow the design of better treatments in the future.

Current alternatives to 5HT3RA include loperamide and the recently introduced eluxadoline. There are only a few very small trials of loperamide in IBS-D, which clearly show its effectiveness in controlling diarrhoea but not pain [31, 32]. Anecdotally, patients often report constipation following loperamide use, and this is associated with bloating and discomfort. Eluxadoline is a combined μ-opioid agonist and δ-opioid antagonist shown to increase the proportion of responders from 5.7% on placebo to 11–13.8% in a dose-response study [33]. However, the main effect was on stool consistency with no obvious effect on pain. Unfortunately, this drug has been associated with acute pancreatitis, which is an unacceptable side effect for most patients with IBS so the search for alternative safe treatments remains important.

If ondansetron is effective in our trial, it could easily be widely adopted since it is an inexpensive, safe, and generic drug. By providing an effective treatment, it could not only reduce patient symptoms, but also reduce healthcare costs associated with repeated referral and unnecessary investigations.

### Protocol version

Protocol version 6.0, 6 November 2018 amended 5 December 2018. Recruitment opened on 19 March 2018, expected completion December 2021.

## Additional file

Additional file 1: SPIRIT 2013 checklist. (DOCX 62 kb)

## Data Availability

Request for access to the data and any material will be considered by the TSC and the co-investigators.

## Abbreviations

5HT: 5-Hydroxytryptamine
5HT_3_RA: 5HT_3_ receptor antagonists
BSFS: Bristol Stool Form Score
CONSORT: Consolidated Standards of Reporting Trials
CTRU: Clinical Trials Research Unit
DMC: Data Monitoring Committee
ECG: Electrocardiogram
FDA: Food and Drug Administration
HADS: Hospital Anxiety and Depression Scale
HAPC: High-amplitude propagating contraction
HRM: High Resolution Manometry
IBS: Irritable bowel syndrome
IBS-D: Irritable bowel syndrome with diarrhoea
IBS-QOL: IBS Quality of Life Questionnaire
IBS-SSS: IBS Severity Scoring System
IMP: Investigational medicinal product
mg: Milligrams
ml: Millilitres
PAR2: Protease activated receptor 2
PHQ-12: Patient Health Questionnaire
PI: Principal Investigator
QT: Interval is the time from the start of the Q wave to the end of the T wave
QTc: Corrected QT
RCT: Randomised controlled trial
SAE: Serious adverse event
SeHCAT: Selenium homocholic acid taurine
SF-LDQ: Short-form Leeds Dyspepsia Questionnaire
t.d.s: Three times a day
TMG: Trial Management Group
TPH-1: Tryptophan hydroxylase 1
TSC: Trial Steering Committee
